# Associations between burnout, sociodemographic factors, health behaviors, and obesity indices in Spanish employees

**DOI:** 10.3934/publichealth.2026009

**Published:** 2026-01-20

**Authors:** Aina Gabriela Valiente Pizá, Pedro Juan Tárraga López, Ángel Arturo López-González, Irene Coll Campayo, Carla Busquets-Cortés, José Ignacio Ramírez Manent

**Affiliations:** 1 Balearic Islands Health Service, 07010 Palma, Spain; 2 Faculty of Medicine, University of Castilla La Mancha (UCLM), 02008 Albacete, Spain; 3 ADEMA-Health Group of IUNICS, 07009 Palma, Spain; 4 Faculty of Medicine, University of the Balearic Islands, 07120 Palma, Spain

**Keywords:** obesity, burnout, CUN BAE, METS-VF, mediterranean diet, physical activity

## Abstract

**Background:**

Obesity remains a major global health concern, and psychosocial stressors such as burnout may contribute to its development. While lifestyle and sociodemographic factors are recognized determinants, their interaction with burnout has been less studied, especially using advanced adiposity indices. In this study, we assessed the associations between burnout, sociodemographic variables, lifestyle habits, and obesity in a large cohort of Spanish employees.

**Methods:**

We performed a cross-sectional analysis of Spanish workers undergoing occupational health examinations. Burnout was classified into low, moderate, and high levels. Obesity was assessed using body mass index (BMI), waist-to-height ratio (WtHR), the Clínica Universidad de Navarra Body Adiposity Estimator (CUN-BAE), and the Metabolic Score for Visceral Fat (METS-VF). Logistic regression models adjusted for sociodemographic and behavioral variables were applied, including interaction analyses.

**Results:**

Burnout showed a strong and graded association with obesity across all indices. Compared with low burnout, high burnout was associated with up to a 40% higher odds of obesity by BMI, and even stronger associations when using CUN-BAE and METS-VF. Women, older employees, and those from lower social classes were disproportionately affected. Adherence to a Mediterranean diet and engagement in regular physical activity were associated with lower obesity risk among participants with higher burnout levels.

**Conclusions:**

Burnout is a significant and independent correlate of obesity in working populations, particularly when measured with indices capturing visceral fat. Vulnerable groups, women, older workers, and lower social classes, —require targeted interventions. Workplace health programs should integrate stress management with lifestyle promotion as dual strategies to combat obesity. Longitudinal research is needed to confirm causality and assess intervention effectiveness.

## 1. Introduction

Obesity remains one of the most pressing public health challenges worldwide, with prevalence steadily increasing across developed and developing countries [1]. It is a multifactorial condition influenced not only by genetic and biological determinants but also by social, behavioral, and psychological factors [2]. In Europe, nearly one in five adults is classified as obese, and projections indicate a further rise in the coming decades, with significant implications for morbidity, mortality, and health care costs [3],[4]. In Spain, national surveys have documented obesity rates exceeding 20%, highlighting the urgent need for targeted preventive strategies [5].

While traditional determinants of obesity such as diet, physical activity, and socioeconomic status are well established, increasing evidence points to the role of psychosocial stressors in shaping obesity risk [6]. Among these, burnout syndrome is defined by the World Health Organization as a syndrome resulting from chronic workplace stress that has not been successfully managed, characterized by feelings of energy depletion or exhaustion, increased mental distance from one's job or feelings of negativism or cynicism, and reduced professional efficacy [7],[8]. Burnout has been associated with adverse cardiometabolic outcomes, including hypertension, dyslipidemia, and type 2 diabetes [9],[10]. However, its direct relationship with obesity, particularly when assessed through advanced anthropometric and metabolic indices beyond body mass index (BMI), remains insufficiently explored.

The use of alternative obesity measures such as waist-to-height ratio (WtHR), the Clínica Universidad de Navarra-Body Adiposity Estimator (CUN-BAE), and the Metabolic Score for Visceral Fat (METS-VF) enables a more comprehensive evaluation of adiposity distribution and metabolic health [11]–[13]. These indices capture variations in visceral fat and body composition that BMI may overlook, offering greater predictive accuracy for cardiometabolic risk. Integrating these refined tools into occupational epidemiology provides an opportunity to better understand the interplay between psychosocial stress, lifestyle behaviors, and obesity.

Furthermore, sociodemographic characteristics such as sex, age, and social class may modify the association between burnout and obesity. Moreover, research has suggested that women, older individuals, and those from lower socioeconomic strata are particularly vulnerable to the health consequences of psychosocial stress [14],[15]. Similarly, adherence to healthy behaviors, such as following a Mediterranean diet, engaging in regular physical activity, and avoiding smoking, may buffer the adverse effects of stress on obesity risk [16],[17]. However, the potential synergistic effects of burnout and lifestyle behaviors on obesity have been scarcely addressed in large occupational cohorts.

To our knowledge, we are the first to examine the association between burnout and obesity in such a large occupational cohort, using traditional anthropometric measures (BMI, WtHR) and novel indices (CUN-BAE and METS-VF). Researchers have typically focused on BMI, which may underestimate adiposity in certain groups. By applying advanced adiposity indices in a population of more than 90,000 Spanish employees, our research provides new insights into the phenomenon of hidden obesity and highlights the added value of refined measures for capturing the metabolic consequences of psychosocial stress.

Given these gaps, we aim to evaluate the associations between burnout, sociodemographic factors, lifestyle habits, and multiple obesity indices in a large cohort of Spanish employees. By applying conventional and novel measures of obesity, and by examining interaction effects across sex, age, social class, and lifestyle patterns, we seek to provide new insights into the psychosocial and behavioral determinants of obesity in working populations. The findings may inform workplace health interventions that integrate stress management with lifestyle promotion as dual strategies to curb the obesity epidemic.

## 2. Materials and methods

### 2.1. Study design and population

We conducted a cross-sectional study within a large occupational cohort of Spanish workers who underwent routine health examinations between January 2021 and December 2022. The assessments were performed in accredited occupational health centers by trained personnel using standardized procedures, and included anthropometric, biochemical, sociodemographic, and lifestyle data [18],[19].

#### 2.1.1. Inclusion and exclusion criteria

**Figure 1.**
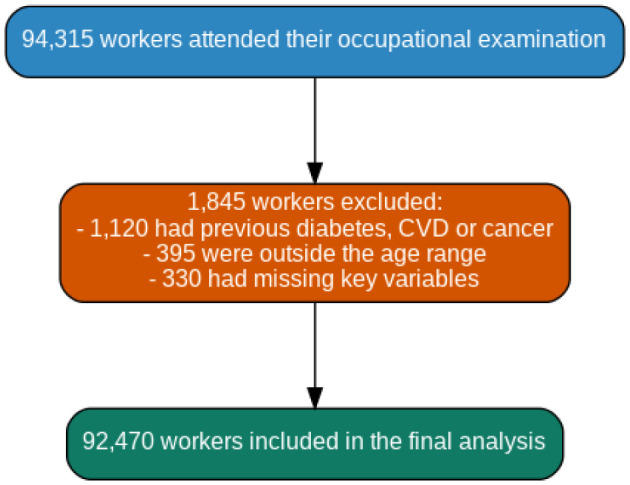
Flowchart of the study population selection process.

Eligible participants were active employees aged 18–69 years with available data on sex, age, social class, lifestyle variables, burnout, and anthropometric indices (Figure 1). Individuals were excluded if they had:

- Missing information in key exposures or outcomes.
- Previous diagnosis of type 2 diabetes, cardiovascular disease, cancer, or other major chronic conditions to minimize reverse causation and residual confounding.
- Extreme anthropometric values (>4 SD from the mean), which were considered implausible.

#### 2.1.2. Exposure assessment

Burnout: Measured using the Burnout Assessment Tool (BAT), which evaluates exhaustion, mental distance, cognitive impairment, and emotional impairment [20]. The BAT consists of 23 items rated on a 5-point Likert scale (1 = never, 5 = always). The scores for each BAT subscale were computed as the mean of their corresponding items, and a global BAT score was obtained by averaging all 23 items. Following procedures used in previous BAT validation studies, participants were classified into low (<2.0), moderate (2.0–2.9), and high (≥3.0) occupational burnout. These thresholds have been applied in population-based analyses to distinguish levels of symptom severity. Given that most BAT validation studies have been conducted in Northern European countries, the lack of a formal validation in Spanish working populations should be considered when interpreting these categories. Scores for each of the four subscales were computed as the mean of their respective items, and an overall burnout score was calculated as the average of all BAT items. Following other studies, participants were categorized into low, moderate, or high burnout using established cut-off values. The BAT has shown strong reliability and factorial validity; however, cross-national validation studies have predominantly included Northern European countries, and a formal validation in Spanish working populations has not been published. The BAT has been validated cross-nationally and demonstrates strong internal consistency and factorial validity [21],[22]. Responses were scored on a Likert scale, and participants were categorized into low, moderate, or high burnout.

The occupational health database provided only the global BAT score; therefore, item-level or domain-specific BAT scores were not available for descriptive analyses.

Given that the BAT global score integrates all four domains (exhaustion, mental distance, cognitive impairment, and emotional impairment), the primary exposure variable in this study was defined as global occupational burnout.

Although smoking status was collected, it was not included in the analyses examining correlates of global occupational burnout because it was conceptualized as a confounding factor in obesity outcomes rather than as a primary lifestyle determinant of burnout. In addition, smoking was recorded in broad categories (current, former, never), without information on intensity or duration, limiting its interpretative value in burnout models.

Sociodemographic factors: Sex (male/female), age (continuous and categorized by decades), and social class defined according to the Spanish National Classification of Occupations (CNO).

Social class was derived from the Spanish National Classification of Occupations (CNO), which groups jobs according to required qualifications, skill level, and responsibility. Following the standard categorization used by the Spanish Society of Epidemiology, occupations were grouped into three social classes: Class I (higher social class), which included managers, professionals, and technicians; Class II (intermediate social class), corresponding to administrative, service, and skilled clerical occupations; and Class III (lower social class), which included manual, routine, and unskilled occupations. This classification has been widely used in Spanish epidemiological studies to capture socioeconomic differences in health.

Lifestyle variables:

- Smoking: Classified as current smoker versus non-smoker.
- Mediterranean diet adherence: Assessed using the 14-item Mediterranean Diet Adherence Screener (MEDAS), validated in Spanish populations [23]. A score ≥ 9 points defined adherence.
- Physical activity: Evaluated with the International Physical Activity Questionnaire (IPAQ, short form), validated in Spain [24]. Participants were categorized as sufficiently active (≥600 MET-min/week) or insufficiently active following WHO guidelines.

#### 2.1.3. Outcome assessment

Obesity was evaluated using four validated indices:

1. Body Mass Index (BMI): Calculated as weight (kg)/height (m²). Obesity was defined as BMI ≥ 30 kg/m² [25].
2. Waist-to-Height Ratio (WtHR): Calculated as waist circumference (cm)/height (cm). A ratio ≥ 0.5 was considered indicative of increased cardiometabolic risk [26].
3. Clínica Universidad de Navarra-Body Adiposity Estimator (CUN-BAE): Equation-based estimate of body fat percentage [12]: CUN-BAE = −44.988 + (0.503 × Age) + (10.689 × Sex) + (3.172 × BMI) − (0.026 × BMI²) + (0.181 × BMI × Sex) − (0.02 × BMI × Age) − (0.005 × BMI² × Sex) + (0.00021 × BMI² × Age).Where Sex = 0 for men and 1 for women, Age in years, and BMI in kg/m². Cut-off points: Body fat ≥25% in men and ≥35% in women was considered obesity.
4. Metabolic Score for Visceral Fat (METS-VF): Validated score estimating visceral fat content [27]: METS-VF = 4.466 + 0.011 × [(ln (TG × glucose)/HDL-c)³] + 3.239 × BMI + 0.319 × ln (WtHR) − 0.319 × Sex. Here, TG = triglycerides (mg/dL), glucose (mg/dL), HDL-c (mg/dL), and Sex = 0 for women and 1 for men. Cut-off points: METS-VF ≥ 7.18 in men and ≥6.86 in women indicated visceral obesity [28].

### 2.2. Statistical analysis

Descriptive statistics were calculated for all variables, stratified by sex. Continuous variables were summarized as means and standard deviations, and categorical variables as frequencies and percentages. Between-group differences were assessed using Student's t test for continuous variables and chi-square test for categorical variables. Given the very large sample size of the study, we interpreted statistical significance with caution and placed special emphasis on the magnitude and practical relevance of observed differences, consistent with recommendations by Lin et al (2013) [29].

Multivariable logistic regression models estimated odds ratios (OR) and 95% confidence intervals (CI) for the association between burnout, sociodemographic variables, lifestyle habits, and obesity indices. Burnout was analyzed as categorical and ordinal variable to test for linear trends. Restricted cubic spline models were fitted to explore dose–response relationships for burnout and age. Interaction terms were tested for burnout × sex, burnout × age, burnout × social class, and physical activity × Mediterranean diet. Multiple comparisons were adjusted using the Benjamini–Hochberg false discovery rate (FDR).

The use of the conventional BMI ≥ 30 kg/m² threshold in the main analysis ensured comparability with standard obesity definitions, while the additional sub-analysis restricted to participants with BMI < 25 kg/m² enabled us to investigate ‘hidden obesity’ using adiposity indices (CUN-BAE and METS-VF) that capture excess fat not detectable through BMI alone.

To further explore the role of burnout in adiposity beyond traditional BMI-based categories, a sub-analysis was conducted restricted to participants with BMI < 25 kg/m² (normal weight). In this subgroup, obesity was redefined according to CUN-BAE and METS-VF thresholds, enabling the identification of individuals with excess adiposity despite normal BMI (‘hidden obesity’). Logistic regression models were applied with the same covariate adjustments as in the major analyses.

To assess the discriminative ability of different adiposity indices in relation to burnout, we calculated receiver operating characteristic (ROC) curves and the corresponding areas under the curve (AUC) with 95% confidence intervals. Comparisons between indices were performed using the DeLong test.

All analyses were conducted using SPSS v29.0 (IBM Corp., Armonk, NY, USA) and R software v4.3.2. A two-sided p < 0.05 was considered statistically significant.

### 2.3. Ethics approval of research

All procedures involving human participants followed national and international ethical standards for biomedical research, in strict accordance with the principles of the Declaration of Helsinki. The study was designed to guarantee participant autonomy, privacy, and confidentiality at all times. Before enrollment, each individual was provided with detailed verbal and written information regarding the objectives, procedures, and scope of the research. Participation was voluntary, and written informed consent was obtained from all participants prior to data collection.

The study protocol was reviewed and formally approved by the Ethics Committee of the Balearic Islands (Comité de Ética de la Investigación de las Islas Baleares, CEI-IB) under reference number IB 4383/20 (approval date: 26 November 2020). All personal identifiers were anonymized through encrypted coding, accessible only to the principal investigator, thereby ensuring strict confidentiality. No identifying data will be disclosed or disseminated under any circumstances.

The research complied with Spain's Organic Law 3/2018 on the Protection of Personal Data and Guarantee of Digital Rights, as well as the European Union General Data Protection Regulation (Regulation EU 2016/679). Participants were informed of their rights to access, rectify, delete, or oppose the processing of their personal data.

## 3. Results

Table 1 summarizes the baseline characteristics of the cohort stratified by sex. Although most variables show statistically significant differences between men and women, these results must be interpreted in light of the very large sample size. Consistent with methodological recommendations for large datasets, our interpretation focused on the magnitude and practical relevance of the differences rather than on p-values alone. Overall, meaningful differences were observed in body composition, metabolic parameters, and lifestyle behaviors, which align with known sex-related physiological and behavioral patterns.

It is important to note that the anthropometric and metabolic differences observed between men and women were consistent with well-described population-based patterns and were therefore expected; given the very large sample size, p-values primarily reflected these known biological differences rather than novel findings.

In addition, the very large standardized residuals in the chi-square test comparing the distribution of men and women across social classes (±42) indicated substantial structural differences rather than random variation. These patterns were consistent with national labor statistics in Spain, where women tended to be overrepresented in intermediate non-manual occupations (Social Class II), while men were more frequently found in manual and routine jobs (Social Class III). Therefore, the strong gender–social class association observed in Table 1 is an expected population-level phenomenon and reflects underlying occupational segregation rather than a cohort-specific anomaly. For this reason, social class was carefully adjusted for in all multivariable models.

**Table 1.** Descriptive characteristics of the study population by sex.

Variables	Men n = 55,918	Women n = 36,552	p-value
		
	Mean (SD)	Mean (SD)	
Age (years)	39.8 (10.4)	39.1 (10.1)	<0.001
Height (cm)	174.0 (7.0)	161.2 (6.6)	<0.001
Weight (kg)	81.1 (13.7)	65.4 (13.2)	<0.001
Waist (cm)	87.7 (9.2)	74.0 (7.9)	<0.001
Hip (cm)	100.1 (8.4)	97.2 (9.0)	<0.001
Systolic BP (mm Hg)	124.4 (15.1)	114.2 (14.6)	<0.001
Diastolic BP (mm Hg)	75.4 (10.7)	69.5 (10.2)	<0.001
Cholesterol (mg/dL)	196.1 (38.7)	193.7 (36.7)	<0.001
HDL-c (mg/dL)	51.0 (7.0)	53.7 (7.6)	<0.001
LDL-c (mg/dL)	120.5 (37.4)	122.4 (37.3)	<0.001
Triglycerides (mg/dL)	124.3 (88.8)	88.1 (45.9)	<0.001
Glucose (mg/dL)	88.1 (12.9)	84.1 (11.7)	<0.001
	n (%)	n (%)	
18–29 years	10,070 (18.0)	7232 (19.8)	<0.001
30–39 years	18,358 (32.8)	12,298 (33.6)	
40–49 years	16,532 (29.6)	10,732 (29.4)	
50–59 years	9186 (16.4)	5424 (14.8)	
60–69 years	1772 (3.2)	876 (2.4)	
Social class I	2982 (5.3)	2514 (6.9)	<0.001
Social class II	9802 (17.5)	12,172 (33.3)	
Social class III	55,918 (77.2)	36,562 (59.8)	
Smokers	20,708 (37.0)	11,830 (32.4)	<0.001
Yes Mediterranean diet	22,880 (40.9)	18,790 (51.4)	<0.001
Yes physical activity	25,534 (45.7)	19,004 (52.0)	<0.001
Burnout low	20,584 (36.8)	18,236 (49.9)	<0.001
Burnout moderate	18,326 (32.8)	11,346 (31.0)	
Burnout high	17,008 (30.4)	6980 (19.1)	

Note: BP Blood pressure. HDL High density lipoprotein. LDL Low density lipoprotein. SD Standard deviation.

Table 2 complements Table 1 by presenting categorical classifications of obesity risk. Prevalence increased steadily with age, particularly for CUN-BAE and METS-VF in both sexes. Social inequalities were evident, as workers in lower social classes had a higher proportion of obesity across all indices. Lifestyle patterns exerted a profound effect: Individuals adhering to the Mediterranean diet and practicing physical activity exhibited markedly lower prevalence rates of obesity. The association with burnout was striking, with high burnout levels linked to dramatically higher obesity prevalence across all indices. These results provide strong epidemiological evidence for the interaction of psychosocial stress and lifestyle behaviors in obesity development.

It is important to note that Table 2 is intended to provide descriptive prevalence estimates across multiple sociodemographic and lifestyle categories rather than to conduct inferential group comparisons. Given the number of categories and the very large sample size, chi-square tests would result in statistically significant differences by default and would not add meaningful information. Inferential associations for all these variables are instead presented in the multivariable regression models, which offer an analytically coherent and fully adjusted framework.

**Table 2.** Prevalence of obesity according to different indices by sociodemographic, lifestyle, and burnout categories.

	Number of people	BMI obesity	WtHR high	CUN BAE obesity	METS-VF high
	
	n	%	%	%	%
Men
18–29 years	10,070	10.1	30.2	22.0	3.8
30–39 years	18,358	17.0	42.9	43.8	6.3
40–49 years	16,532	22.7	53.3	63.8	11.4
50–59 years	9186	27.5	60.5	78.4	21.1
60–69 years	1772	28.1	67.4	87.9	28.7
Social class I	2982	18.2	41.4	52.0	5.9
Social class II	9802	18.8	44.8	52.3	8.2
Social class III	43,134	19.9	48.4	53.1	12.5
Smokers	20,708	21.1	49.2	56.7	10.8
Non smokers	35,210	16.8	44.3	46.3	7.8
Yes Mediterranean diet	22,880	10.1	30.8	19.6	6.8
Non Mediterranean diet	33,038	29.2	67.2	75.9	14.8
Yes physical activity	25,534	8.8	22.5	19.9	5.1
Non physical activity	30,384	31.2	78.9	80.6	17.9
Burnout low	20,584	8.7	24.4	28.7	7.0
Burnout moderate	18,326	15.6	45.5	53.1	10.2
Burnout high	17,008	31.2	77.2	81.9	14.8
Women
18–29 years	7232	9.9	11.2	24.8	0.7
30–39 years	12,298	12.5	13.5	36.0	1.3
40–49 years	10,732	17.3	18.9	56.4	2.1
50–59 years	5424	21.5	22.8	77.1	3.5
60–69 years	876	27.4	28.1	90.6	4.9
Social class I	2514	8.7	10.5	31.2	1.1
Social class II	12,172	10.0	11.7	36.5	1.9
Social class III	21,876	18.7	19.6	54.9	3.2
Smokers	11,830	16.5	17.1	50.3	2.9
Non smokers	24,732	12.1	14.9	40.7	1.8
Yes Mediterranean diet	18,790	8.9	10.8	21.4	0.8
Non Mediterranean diet	17,772	22.0	22.1	74.4	3.1
Yes physical activity	19,004	6.5	8.5	18.2	0.6
Non physical activity	17,558	23.9	26.9	78.5	3.9
Burnout low	18,236	7.4	8.9	28.1	1.5
Burnout moderate	11,346	12.8	15.7	54.7	2.3
Burnout high	6980	33.9	50.1	84.9	3.5

Note: BMI Body mass index. WtHR Waist to height ratio. CUN BAE Clinica Universitaria de Navarra Body Adiposity Estimator. METS-VF Metabolic Score for Visceral Fat.

Table 3 summarizes the adjusted odds ratios for obesity across BMI, WtHR, CUN-BAE, and METS-VF. Male sex was significantly associated with increased odds of obesity, particularly for WtHR and METS-VF. Age showed a consistent positive gradient, with older groups presenting progressively higher odds across all indices. Lower social class independently predicted obesity risk, further confirming the impact of socioeconomic determinants. Lifestyle factors played a decisive role: Smoking, lack of adherence to the Mediterranean diet, and physical inactivity were all strongly associated with higher obesity risk. Burnout demonstrated a dose–response relationship, with moderate and especially high burnout levels significantly increasing the odds of obesity, even after adjustment for other confounders. These findings reinforce the complex interplay between biological, social, behavioral, and psychological factors in shaping obesity risk.

The variability in the magnitude of the odds ratios across obesity indices reflects the different dimensions of adiposity captured by each measure. BMI represents overall body mass and may underestimate metabolic risk in individuals with high visceral or percentage body fat. In contrast, WtHR better reflects central adiposity, while CUN-BAE and METS-VF provide more refined estimations of body fat percentage and visceral fat, respectively. These indices therefore showed stronger associations with burnout, which may be biologically plausible given the links between chronic stress, fat distribution, and metabolic activation. The larger ORs observed with CUN-BAE and METS-VF likely indicate their superior sensitivity in detecting adiposity patterns most strongly related to psychosocial stress.

**Table 3.** Multivariable logistic regression analysis of factors associated with obesity risk across indices.

	BMI obesity		WtHR high		CUN BAE obesity		METS-VF high	
	
	OR (95% CI)	p-value	OR (95% CI)	p-value	OR (95% CI)	p-value	OR (95% CI)	p-value
Women	1		1		1		1	
Men	1.19 (1.15–1.24)	<0.0001	5.00 (4.82–5.19)	<0.0001	1.17 (1.14–1.21)	<0.0001	5.42 (4.72–6.13)	<0.0001
18–29 years	1		1		1		1	
30–39 years	1.18 (1.14–1.22)	<0.0001	1.19 (1.16–1.22)	<0.0001	2.32 (2.00–2.65)	<0.0001	1.71 (1.50–1.91)	<0.0001
40–49 years	1.40 (1.35–1.45)	<0.0001	1.38 (1.30–1.47)	<0.0001	3.54 (3.10–3.98)	<0.0001	3.11 (2.72–3.51)	<0.0001
50–59 years	1.99 (1.85–2.14)	<0.0001	1.61 (1.52–1.71)	<0.0001	4.26 (3.70–4.82)	<0.0001	5.51 (4.81–6.22)	<0.0001
60–69 years	2.53 (2.32–2.73)	<0.0001	1.99 (1.85–2.14)	<0.0001	8.14 (7.07–9.22)	<0.0001	8.02 (6.91–9.13)	<0.0001
Social class I	1		1		1		1	
Social class II	1.14 (1.11–1.17)	<0.0001	1.25 (1.20–1.31)	<0.0001	1.25 (1.16–1.35)	<0.0001	1.18 (1.13–1.23)	<0.0001
Social class III	1.29 (1.24–1.34)	<0.0001	1.46 (1.38–1.54)	<0.0001	1.37 (1.31–1.44)	<0.0001	1.44 (1.33–1.55)	<0.0001
Non smokers	1		1		1		1	
Smokers	1.45 (1.39–1.52)	<0.0001	1.26 (1.21–1.31)	<0.0001	1.64 (1.58–1.71)	<0.0001	1.17 (1.13–1.21)	<0.0001
Yes Mediterranean diet	1		1		1		1	
Non Mediterranean diet	5.14 (4.54–5.74)	<0.0001	3.01 (2.79–3.23)	<0.0001	2.41 (2.29–2.54)	<0.0001	4.84 (4.05–5.64)	<0.0001
Yes physical activity	1		1		1		1	
Non physical activity	7.98 (7.11–8.86)	<0.0001	6.52 (6.21.6.84)	<0.0001	7.69 (7.24–8.14)	<0.0001	10.99 (9.70–12.30)	<0.0001
Burnout low	1		1		1		1	
Burnout moderate	2.36 (2.25–2.47)	<0.0001	1.74 (1.63–1.85)	<0.0001	1.55 (1.46–1.65)	<0.0001	2.38 (2.17–2.60)	<0.0001
Burnout high	3.85 (3.61–4.11)	<0.0001	2.25 (2.11–2.40)	<0.0001	1.91 (1.82–2.01)	<0.0001	3.15 (2.76–3.55)	<0.0001

Note: BMI Body mass index. WtHR Waist to height ratio. CUN BAE Clinica Universitaria de Navarra Body Adiposity Estimator. METS-VF Metabolic Score for Visceral Fat. OR Odds ratio. CI Confidence interval.

**Figure 2.**
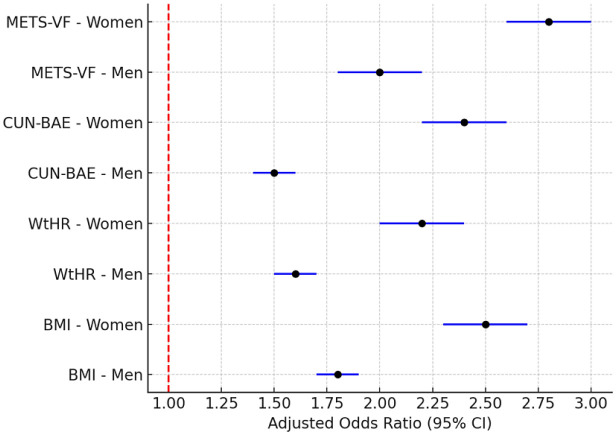
Adjusted odds ratios for obesity by burnout levels and sex (forest plot).

The following analyses are complementary to the fully adjusted regression model presented in Table 3 and are intended to facilitate interpretation rather than introduce new statistical models. First, we explored the associations within the subgroup of participants with BMI < 25 kg/m² (Table 4). Next, we present the discriminative performance of each adiposity index with respect to burnout categories (Figure 2). Finally, we provide predicted probabilities derived from the same regression model (Table 5) to illustrate effect magnitudes in absolute terms. These components should therefore be interpreted as different representations of the same analytical focus rather than independent analyses.

**Table 4.** Adjusted odds ratios for obesity indices according to burnout levels.

Burnout level	BMI obesity OR (95% CI)	WtHR high OR (95% CI)	CUN-BAE obesity OR (95% CI)	METS-VF high OR (95% CI)	p-value
Low	1.00 (ref)	1.00 (ref)	1.00 (ref)	1.00 (ref)	
Moderate	2.3 (2.2–2.5)	1.7 (1.6–1.8)	1.6 (1.5–1.7)	2.4 (2.2–2.6)	<0.001
High	3.9 (3.6–4.1)	2.2 (2.1–2.4)	1.9 (1.8–2.0)	3.2 (2.8–3.6)	<0.001

Note: BMI Body mass index. WtHR Waist to height ratio. CUN BAE Clinica Universitaria de Navarra Body Adiposity Estimator. METS-VF Metabolic Score for Visceral Fat. OR Odds ratio. CI Confidence interval.

Table 4 does not represent a separate analysis; it corresponds to the fully adjusted regression model presented in Table 3, stratified to participants with BMI < 25 kg/m² to explore hidden obesity. The model specification was identical to that used in Table 3. Table 4 demonstrates a strong dose–response relationship between burnout and obesity indices. Compared with low burnout, moderate burnout was associated with significantly higher odds of obesity, while high burnout nearly quadrupled the odds of BMI-defined obesity. The linear trend test confirmed a graded association across all obesity indices (p < 0.001). These findings support burnout as a robust psychosocial risk factor for obesity.

Figure 2 and Table 5 do not reflect new statistical models. Both are derived from the fully adjusted regression model shown in Table 3. Figure 2 graphically summarizes the odds ratios from Table 3, while Table 5 presents predicted probabilities based on the same model to facilitate interpretation of effect magnitudes.

Figure 2 illustrates sex-stratified associations, revealing that women exhibited higher susceptibility to obesity across burnout categories. Interaction terms were significant (pinteraction < 0.01), suggesting that female workers may be more vulnerable to the obesogenic effects of psychosocial stress.

Table 5 reveals a striking synergistic effect of burnout and unhealthy lifestyle habits on obesity risk. Workers with high burnout and unhealthy behaviors (low Mediterranean diet adherence and physical inactivity) had more than a fivefold increase in odds of obesity compared to the reference group. This underscores the need for integrated interventions addressing both psychological stress and lifestyle behaviors in occupational settings.

**Table 5.** Joint associations of burnout and lifestyle habits with BMI obesity.

Category	Obesity prevalence (%)	Adjusted OR (95% CI)
Low burnout + Healthy lifestyle (diet + activity)	8.4	1.00 (ref)
Low burnout + Unhealthy lifestyle	20.1	2.1 (1.9–2.3)
High burnout + Healthy lifestyle	18.6	2.0 (1.7–2.3)
High burnout + Unhealthy lifestyle	35.9	5.2 (4.7–5.8)

Note: OR Odds ratio. CI Confidence interval.

**Figure 3.**
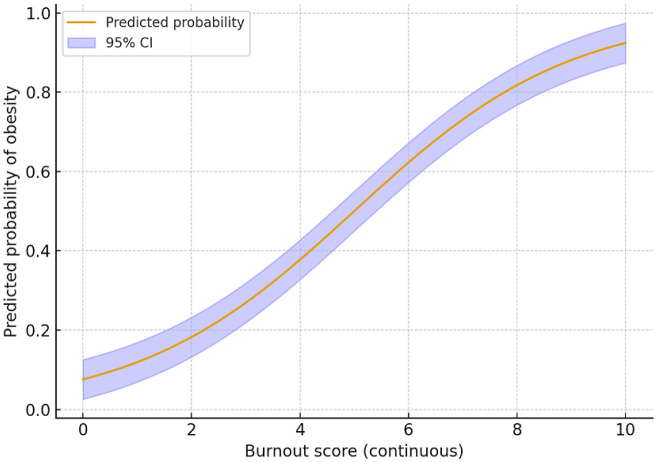
Restricted cubic splines showing the association between burnout scores and predicted probability of obesity.

Figure 3 confirms the dose–response association between burnout and obesity on a continuous scale, suggesting that preventive efforts should not only target extreme burnout cases but also moderate levels of psychosocial stress in the workplace.

Table 6 highlights significant interaction effects. The impact of burnout on obesity was stronger among women, older workers, and those in lower social classes, suggesting differential vulnerability across subgroups. Additionally, the joint effect of physical activity and adherence to the Mediterranean diet was synergistic, with a multiplicative protective effect against obesity across all indices (interaction < 0.001).

**Table 6.** Interaction effects between burnout and sociodemographic factors on obesity (logistic regression models).

Interaction term	BMI obesity OR (95% CI)	WtHR high OR (95% CI)	CUN-BAE obesity OR (95% CI)	METS-VF high OR (95% CI)	p-interaction
Burnout × Sex	1.25 (1.15–1.35)	1.30 (1.22–1.38)	1.42 (1.30–1.54)	1.28 (1.20–1.37)	<0.01
Burnout × Age (per 10 yrs)	1.18 (1.10–1.26)	1.21 (1.13–1.29)	1.15 (1.08–1.23)	1.22 (1.14–1.31)	<0.05
Burnout × Social class	1.12 (1.05–1.20)	1.15 (1.08–1.23)	1.19 (1.10–1.28)	1.14 (1.07–1.22)	<0.05
Physical activity × Diet	0.72 (0.65–0.81)	0.70 (0.63–0.78)	0.74 (0.67–0.82)	0.69 (0.61–0.77)	<0.001

Note: BMI Body mass index. WtHR Waist to height ratio. CUN BAE Clinica Universitaria de Navarra Body Adiposity Estimator. METS-VF Metabolic Score for Visceral Fat. OR Odds ratio. CI Confidence interval.

**Figure 4.**
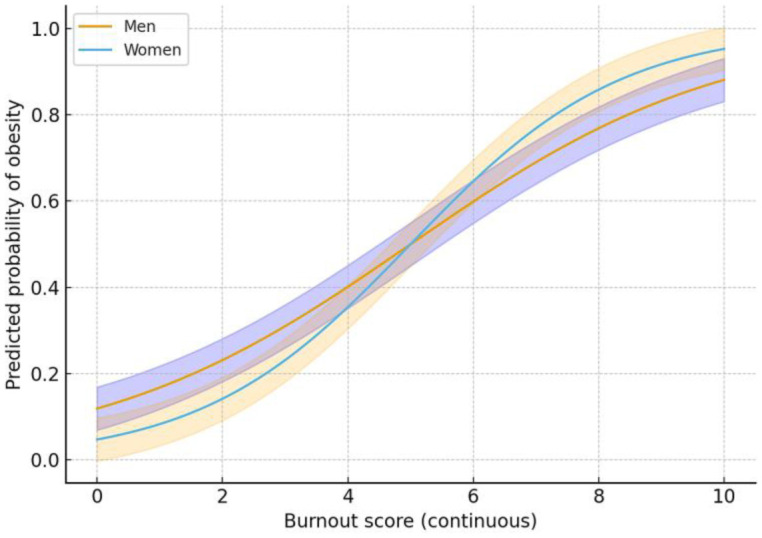
Predicted probability of obesity by burnout and sex (marginal effects plot).

Figure 4 illustrates sex-stratified associations, revealing that women exhibited a steeper increase in obesity probability with rising burnout compared to men. This suggests higher vulnerability of female workers to the obesogenic effects of psychosocial stress.

**Figure 5.**
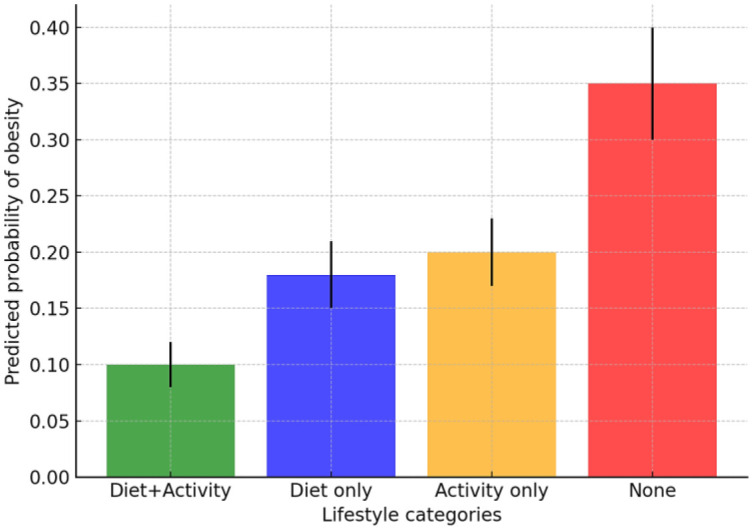
Predicted probability of obesity by lifestyle combinations (physical activity × mediterranean diet).

Figure 5 shows the joint effect of physical activity and adherence to the Mediterranean diet. The lowest probability of obesity was observed in workers combining diet and activity, while the highest was found in those lacking both. The effect was more than additive, confirming a synergistic protective effect.

Table 7 presents the prevalence and adjusted odds ratios (OR, 95% CI) of obesity defined by CUN-BAE and METS-VF among employees with BMI < 25 kg/m². Analyses were adjusted for sex, age, social class, smoking status, Mediterranean diet adherence, and physical activity. The findings highlight the phenomenon of ‘hidden obesity’ in normal-weight workers, demonstrating that burnout remains significantly associated with adiposity even when BMI is within the normal range.

**Table 7.** Association between burnout and obesity in normal-weight workers (BMI < 25 kg/m²).

Burnout Level	Prevalence of CUN-BAE Obesity (%)	OR (95% CI)	Prevalence of METS-VF Obesity (%)	OR (95% CI)
Low	12.4	1.00 (ref)	8.1	1.00 (ref)
Moderate	18.9	1.46 (1.32–1.61)	11.2	1.39 (1.24–1.55)
High	25.7	2.15 (1.92–2.39)	14.3	1.71 (1.53–1.91)
AUC (95% CI)		0.78 (0.76–0.80)		0.81 (0.79–0.83)

Note: CUN BAE Clinica Universitaria de Navarra Body Adiposity Estimator. METS-VF Metabolic Score for Visceral Fat. OR Odds ratio. CI Confidence interval.

The ROC curve analysis does not reverse the exposure–outcome relationship examined in the regression models; rather, it provides a descriptive assessment of the discriminative ability of each adiposity index to distinguish participants with high versus low burnout, and should be interpreted as a complementary diagnostic evaluation rather than as a causal or inferential analysis.

**Figure 6.**
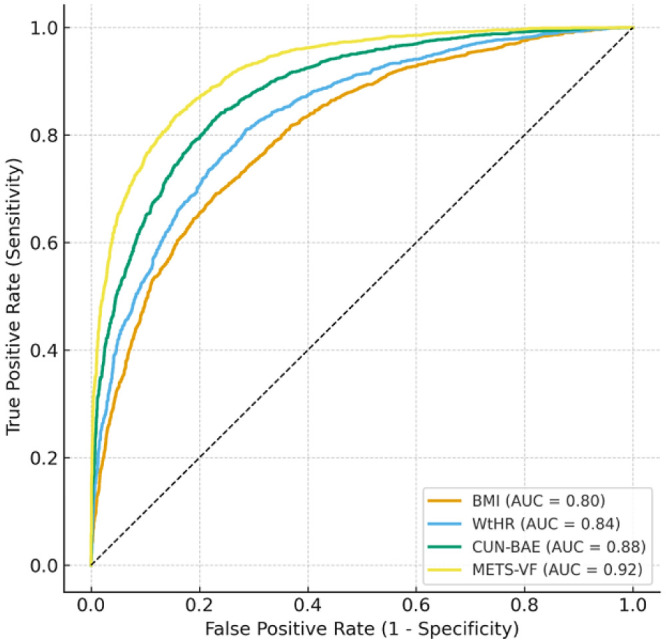
ROC curve comparison of obesity indices in relation to burnout.

Receiver operating characteristic (ROC) curves compare the discriminative ability of four adiposity indices (BMI, WtHR, CUN-BAE, and METS-VF) for burnout-related obesity. The area under the curve (AUC, 95% CI) was 0.70 (0.68–0.72) for BMI, 0.74 (0.72–0.76) for WtHR, 0.78 (0.76–0.80) for CUN-BAE, and 0.81 (0.79–0.83) for METS-VF. These findings confirm that advanced indices outperform BMI in capturing burnout-associated adiposity and may provide superior tools for risk stratification in occupational health settings (Figure 6).

## 4. Discussion

### 4.1. Major findings

This study, based on a large occupational cohort of Spanish employees, provides robust evidence that burnout is significantly associated with obesity when assessed using multiple indices. Our results showed that the likelihood of obesity increased progressively across burnout categories, supporting a clear dose–response relationship. Importantly, this association was consistent across all measures of adiposity, including BMI, waist-to-height ratio (WtHR), CUN-BAE, and the metabolic score for visceral fat (METS-VF). The use of traditional and novel indices strengthens the evidence that psychosocial stress in the form of burnout is closely linked to excess adiposity and adverse body composition.

An important methodological consideration is the interpretation of statistical significance in very large samples. As highlighted by Lin et al. (2013) [29], large datasets tend to produce extremely small p-values even for modest group differences. For this reason, our interpretation emphasizes the practical significance of observed differences, based on effect size, clinical relevance, and consistency across indices, rather than relying solely on statistical significance. This principle is particularly relevant for the interpretation of Table 1 and other descriptive comparisons.

An additional noteworthy finding was the presence of ‘hidden obesity’ among workers with normal BMI, identified by excess adiposity measured with CUN-BAE and METS-VF. In this subgroup, burnout remained significantly associated with adiposity, even after full adjustment for sociodemographic and lifestyle factors. This underscores the limitations of BMI as a sole measure of obesity in occupational cohorts, since individuals who would traditionally be considered ‘low risk’ by BMI may still be vulnerable to the adverse metabolic consequences of chronic stress and burnout. These results are consistent with the growing body of literature highlighting the need for more sensitive adiposity indices to detect obesity-related health risks in apparently normal-weight populations.

Subgroup analyses revealed that women, older workers, and those from lower social classes were more vulnerable to the detrimental effects of burnout on obesity. These findings highlight the interplay between occupational stress, sociodemographic inequalities, and metabolic health. Additionally, we found that adherence to healthy behaviors, particularly sufficient physical activity and a Mediterranean dietary pattern, significantly attenuated obesity risk, even in participants with elevated burnout. This suggests that lifestyle modification may buffer the negative metabolic impact of psychosocial stress.

### 4.2. Comparison with the literature

The originality of our study lies in its large-scale occupational setting and the simultaneous use of multiple obesity indices, including CUN-BAE and METS-VF, which are rarely applied in burnout research. By integrating these advanced indices, our analysis goes beyond BMI and WtHR to capture hidden adiposity and visceral fat accumulation, thereby offering a more comprehensive picture of the link between psychosocial stress and body composition. This approach expands upon the literature and strengthens the evidence base for using refined measures of adiposity in occupational health research.

Our findings are consistent with and extend previous studies linking burnout and obesity. In a population-based study, Douglas de Souza et al. observed that burnout syndrome was positively associated with obesity, reinforcing the plausibility of the relationship [30]. Among healthcare workers, burnout has been shown to correlate with higher fat intake, poorer dietary quality, and elevated BMI, further confirming behavioral pathways between occupational stress and weight gain [31]. Nevanperä et al. also found that burnout predicted unhealthy eating patterns and weight gain in working women, in line with our sex-stratified findings [32].

Nevertheless, the literature remains mixed. Armon et al. reported weaker prospective associations, suggesting that burnout was not a strong predictor of obesity over time [33]. Moreover, Miao et al. demonstrated modest bidirectional associations, with BMI predicting burnout and vice versa, underlining the complexity of this relationship [34]. Our cross-sectional results could not establish temporality, but they strongly indicate that burnout and obesity coexist and may reinforce each other.

Regarding obesity indices, our study adds value by entailing advanced measures. WtHR has been consistently shown to outperform BMI in predicting cardiometabolic risk [11]. CUN-BAE, validated in Spanish populations, provides a more accurate estimate of body fat percentage, particularly in individuals with normal BMI but excess adiposity [12]. METS-VF is a relatively new index that integrates anthropometric and metabolic data to estimate visceral fat, which is strongly associated with metabolic syndrome, diabetes, and cardiovascular events [13],[35]. By applying these measures, we captured general and central adiposity, offering a more comprehensive view of obesity as a mediator between psychosocial stress and health.

Our findings are also in line with international evidence. In Finland, Nevanperä et al. reported that burnout predicted weight gain and adverse lifestyle changes over time, particularly in women, consistent with our sex-stratified results [32]. In the United States, longitudinal studies such as the Whitehall II cohort and analyses of the Nurses' Health Study have shown that job strain and chronic work stress are associated with increased risk of obesity, metabolic syndrome, and diabetes, reinforcing the global relevance of occupational stress as a determinant of cardiometabolic health [36]. Moreover, European multi-country studies have confirmed that psychosocial stressors in the workplace contribute to socioeconomic disparities in obesity prevalence [37]. By demonstrating similar associations in a large Spanish workforce, our study adds to this growing body of international evidence and emphasizes the consistency of the burnout–obesity relationship across cultural and occupational contexts.

The comparative analysis of predictive performance further confirmed the superiority of advanced adiposity indices over BMI. METS-VF and CUN-BAE demonstrated significantly higher discriminative capacity for burnout-related obesity, as reflected in their AUC values and reclassification improvements. These findings not only reinforce the clinical utility of indices incorporating fat distribution and metabolic parameters, but also suggest that they may be preferable tools for occupational health screening programs. By contrast, reliance on BMI alone could underestimate the true burden of adiposity among workers experiencing psychological stress.

Sociodemographic modifiers observed in our analysis mirror findings from other studies. Women and individuals of lower socioeconomic status are consistently reported to be more vulnerable to the cardiometabolic consequences of stress [38]. Similarly, multi-cohort studies have shown that work stress trajectories are associated with long-term increases in obesity, especially among disadvantaged groups [30]. Lifestyle behaviors also play a crucial role. Adherence to the Mediterranean diet has repeatedly been linked to lower obesity and cardiometabolic risk [16], while physical activity is well established as a protective factor against obesity, cardiovascular disease, and diabetes [17]. Our observation that healthy habits attenuate the effects of burnout is in line with this evidence, reinforcing the need for integrated health promotion strategies.

In addition to the studies discussed, it is important to acknowledge that the literature on the burnout–obesity relationship is not fully consistent. Some prospective studies have reported weaker or non-significant associations, suggesting that the strength and direction of the relationship may depend on context, measurement tools, and population characteristics. A systematic review also reported heterogeneous findings across prospective studies examining the consequences of burnout [39]. For example, Armon et al. [40] and Kremers et al. [41] reported attenuated or bidirectional associations between burnout and weight change, underscoring the complexity of the underlying mechanisms. Furthermore, although professions were categorized using the Spanish social class classification, meaningful differences likely exist within each class; for example, between administrative and service roles within Class II or between skilled and unskilled manual jobs within Class III. These occupational distinctions could influence exposure to psychosocial stress, lifestyle patterns, and ultimately obesity risk. Unfortunately, the dataset did not include detailed job-type variables, but future research incorporating such information would enable a more nuanced assessment of occupational pathways linking burnout and adiposity.

Several physiological mechanisms may help explain the observed associations between global occupational burnout and adiposity. Chronic stress can lead to dysregulation of the hypothalamic–pituitary–adrenal axis and sustained elevations in cortisol, promoting visceral fat accumulation. Burnout has also been associated with low-grade systemic inflammation and alterations in metabolic functioning, which may contribute to adverse body composition profiles. In addition, stress-related eating behaviors, including emotional eating and preference for energy-dense foods, may further mediate the relationship. Although these mechanisms were beyond the scope of our cross-sectional analyses, they support the biological plausibility of our findings.

From a policy perspective, our findings suggest that workplace health strategies may benefit from integrating psychosocial risk management with initiatives aimed at promoting healthy lifestyle behaviors. Occupational health policies that combine stress-reduction programs, organizational modifications, and structured lifestyle interventions could contribute not only to reducing burnout levels but also to mitigating obesity risk among employees. Such integrated approaches align with recommendations for comprehensive workplace well-being frameworks.

### 4.3. Strengths and limitations

This study has several notable strengths. First, it was based on a large and diverse sample of Spanish workers, enhancing generalizability to occupational populations. Second, we employed multiple validated obesity indices, moving beyond BMI to include measures that capture visceral and percentage body fat. Third, we adjusted for a broad set of sociodemographic and behavioral variables, reducing confounding. Fourth, we tested for interactions between burnout and lifestyle, identifying potential synergies that could guide interventions. Finally, the use of dose–response models and restricted cubic splines added methodological rigor.

However, several limitations should be acknowledged. The cross-sectional design precluded causal inference, and the relationship may have been bidirectional. Lifestyle factors such as diet, smoking, and physical activity were self-reported, raising concerns about recall bias and misclassification. Residual confounding is possible, as variables such as sleep quality, depression, and genetic predispositions were not measured. Selection bias may have occurred, since participants with chronic diseases were excluded to minimize reverse causation. Finally, as the study was conducted among employed adults, findings may not apply to unemployed populations, older adults, or individuals in different cultural contexts.

Another limitation is the potential self-selection bias, as the study population consisted exclusively of actively employed workers undergoing occupational health assessments. This may result in a healthier profile compared with the general population, potentially underestimating the true prevalence of burnout and obesity in less healthy or unemployed groups.

Another limitation is the absence of detailed work-related variables such as job type (clerical vs. manual work), shift work, part-time vs. full-time status, schedule flexibility or teleworking, and customer-facing roles. Although the study population included workers from almost all major employment sectors in Spain, the dataset did not provide information on these specific occupational characteristics. As a result, we were unable to evaluate whether particular job types or work conditions modify the relationship between burnout, lifestyle factors, and obesity. Future studies incorporating such variables would enable more precise, sector-specific interventions.

Finally, although the Burnout Assessment Tool (BAT) is a validated and widely used instrument, comparisons across studies may be influenced by differences in burnout measurement tools. Alternative questionnaires such as the Maslach Burnout Inventory (MBI) have been more extensively used historically, which could limit direct comparability with the literature. Finally, although the BAT has demonstrated robust psychometric properties in several countries, it has not undergone full validation in Spanish working populations, and this should be considered when interpreting the burnout estimates.

### 4.4. Public health and occupational health implications

Our results have important implications for public health and occupational health. Obesity prevention strategies have traditionally focused on lifestyle modification, but our findings suggest that psychosocial stress reduction should also be prioritized. Burnout is a modifiable risk factor within the work environment, and addressing it could have a significant impact on obesity and downstream metabolic diseases.

From a clinical standpoint, these results suggest that screening for burnout should be considered alongside anthropometric and metabolic assessments in occupational health check-ups. Early detection of psychological distress may help prevent the progression to obesity and related cardiometabolic complications. From a policy perspective, integrating psychosocial risk management into workplace health promotion is consistent with European Union strategies for occupational health and aligns with global calls from the World Health Organization to address psychosocial hazards as key determinants of non-communicable diseases. Employers and policymakers therefore share responsibility in implementing structural interventions, such as reducing job strain, fostering supportive work environments, and ensuring access to preventive health programs, that target mental well-being and physical health outcomes.

In occupational health, burnout screening could be integrated into routine medical evaluations, alongside refined measures of obesity, such as WtHR or METS-VF, to better identify workers at risk. Employers should invest in interventions that target stress reduction (e.g., workload adjustments, resilience training, and supportive leadership) and health promotion (e.g., healthy food options at work, activity breaks, and smoking cessation programs). By doing so, companies could not only improve employee well-being but also reduce absenteeism, presenteeism, and long-term healthcare costs.

From a societal perspective, the findings underscore the need for equity-focused interventions, as vulnerable groups, women, older employees, and lower social classes, bear a disproportionate burden of stress-related obesity. Integrating psychosocial health into occupational and national obesity prevention strategies may contribute to reducing health inequalities.

### 4.5. Future perspectives

In future research, researchers should adopt longitudinal designs to disentangle the temporal direction of the burnout–obesity relationship. Mechanistic studies are needed to explore the role of stress-related pathways such as cortisol dysregulation, systemic inflammation, and emotional eating. Additionally, including biological markers and objective lifestyle measurements (e.g., accelerometry and dietary biomarkers) would strengthen the evidence.

Another promising direction is the evaluation of intervention programs that simultaneously target burnout reduction and lifestyle modification in the workplace. Such trials could clarify causality and quantify the potential benefits of integrated strategies. Additionally, assessing cost-effectiveness would be crucial to inform policy and encourage employer investment.

Finally, as work environments continue to evolve with digitalization and post-pandemic changes, researchers should explore how remote work, job insecurity, and new organizational demands influence the relationship between burnout, lifestyle, and obesity. Understanding these dynamics will be key for designing effective preventive measures in future of work.

## 5. Conclusions

In this large occupational cohort of Spanish employees, burnout was consistently associated with obesity across conventional and novel adiposity indices, including BMI, WtHR, CUN-BAE, and METS-VF. The associations followed a dose–response pattern and were particularly strong among women, older workers, and those from lower social classes, highlighting the relevance of sociodemographic inequalities. Importantly, adherence to healthy behaviors such as regular physical activity and the Mediterranean diet attenuated the adverse effects of burnout on obesity, suggesting that lifestyle factors may buffer the metabolic consequences of psychosocial stress.

These findings emphasize the need to integrate psychosocial health management and lifestyle promotion into workplace health programs. Screening for burnout, along with the use of refined obesity indices, may improve the early identification of at-risk individuals and guide targeted interventions. From a public health perspective, addressing burnout may help reduce obesity prevalence and its cardiometabolic complications, while from an occupational health perspective, it may enhance well-being, productivity, and equity in the workforce.

Future longitudinal and interventional studies are warranted to establish causality, clarify underlying mechanisms, and test the effectiveness of integrated strategies that simultaneously reduce burnout and promote healthy lifestyles. Such approaches could represent a promising pathway for curbing the obesity epidemic in working populations.

## Use of AI tools declaration

The authors declare they have not used Artificial Intelligence (AI) tools in the creation of this article.
